# Processes and mechanisms of coastal woody‐plant mortality

**DOI:** 10.1111/gcb.16297

**Published:** 2022-07-29

**Authors:** Nate G. McDowell, Marilyn Ball, Ben Bond‐Lamberty, Matthew L. Kirwan, Ken W. Krauss, J. Patrick Megonigal, Maurizio Mencuccini, Nicholas D. Ward, Michael N. Weintraub, Vanessa Bailey

**Affiliations:** ^1^ Atmospheric Sciences and Global Change Division Pacific Northwest National Lab Richland Washington USA; ^2^ School of Biological Sciences Washington State University Pullman Washington USA; ^3^ Plant Science Division, Research School of Biology The Australian National University Acton Australian Capital Territory Australia; ^4^ Joint Global Change Research Institute, Pacific Northwest National Laboratory College Park Maryland USA; ^5^ Virginia Institute of Marine Science, William & Mary Gloucester Point Virginia USA; ^6^ U.S. Geological Survey, Wetland and Aquatic Research Center Lafayette Louisiana USA; ^7^ Smithsonian Environmental Research Center Edgewater Maryland USA; ^8^ ICREA, Passeig Lluís Companys 23 Barcelona Spain; ^9^ CREAF Campus UAB, Bellaterra Barcelona Spain; ^10^ Marine and Coastal Research Laboratory Pacific Northwest National Laboratory Sequim Washington USA; ^11^ School of Oceanography University of Washington Seattle Washington USA; ^12^ Department of Environmental Sciences University of Toledo Toledo Ohio USA; ^13^ Biological Sciences Division Pacific Northwest National Laboratory Washington USA

**Keywords:** carbon starvation, coastal, climate change, hydraulic failure, hypoxia, mortality, salinity, sea level rise

## Abstract

Observations of woody plant mortality in coastal ecosystems are globally widespread, but the overarching processes and underlying mechanisms are poorly understood. This knowledge deficiency, combined with rapidly changing water levels, storm surges, atmospheric CO_2_, and vapor pressure deficit, creates large predictive uncertainty regarding how coastal ecosystems will respond to global change. Here, we synthesize the literature on the mechanisms that underlie coastal woody‐plant mortality, with the goal of producing a testable hypothesis framework. The key emergent mechanisms underlying mortality include hypoxic, osmotic, and ionic‐driven reductions in whole‐plant hydraulic conductance and photosynthesis that ultimately drive the coupled processes of hydraulic failure and carbon starvation. The relative importance of these processes in driving mortality, their order of progression, and their degree of coupling depends on the characteristics of the anomalous water exposure, on topographic effects, and on taxa‐specific variation in traits and trait acclimation. Greater inundation exposure could accelerate mortality globally; however, the interaction of changing inundation exposure with elevated CO_2_, drought, and rising vapor pressure deficit could influence mortality likelihood. Models of coastal forests that incorporate the frequency and duration of inundation, the role of climatic drivers, and the processes of hydraulic failure and carbon starvation can yield improved estimates of inundation‐induced woody‐plant mortality.

## Graphical abstract

Coastal woody plant mortality is rising but the underlying mechanisms are poorly understood. Here we summarize what is known regarding the mechanisms of coastal plant mortality and generate a testable framework of hypotheses. This framework provides a roadmap for future empirical and modeling research.

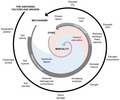

## 1 INTRODUCTION

**FIGURE 1:**
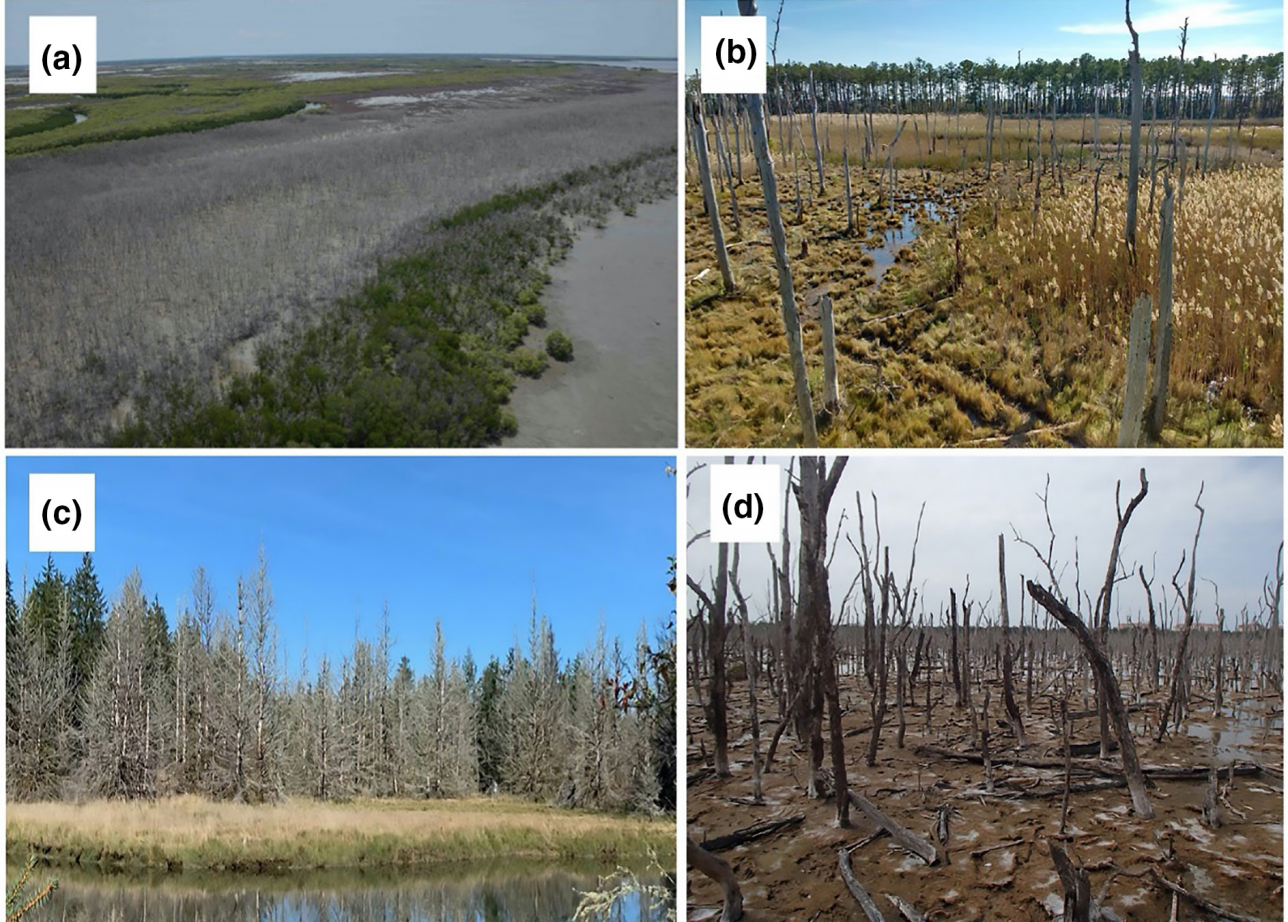
Woody‐plant mortality in coastal ecosystems leads to large‐scale ghost forest formation globally. (a) Mangrove (*Avicennia marina* and *Rhizophora stylosa*) mortality in Australia (N. Duke). (b) Loblolly pine (*Pinus taeda*) mortality, Maryland, USA (M. Kirwan). (c) Sitka spruce (*Picea sitchensis*) mortality, Washington, USA (Nicholas Ward). (d) Mangrove (*Avicennia germinans*) mortality in Florida, USA (Ken Krauss).

Woody‐plant dominated coastal ecosystems are showing increasing evidence of large‐scale mortality (Sippo et al., 2018). Observations of ‘ghost forest’ formation from inundation are becoming globally widespread for both halophytic and glycophytic woody plants (Figure 1; Table 1; Jimenez et al., 1985; Kirwan & Gedan, 2019; Lovelock et al., 2017; Penfound & Hathaway, 1938; Shreve et al., 1910; Sippo et al., 2018; Wang et al., 2019). Accelerating relative sea‐level rise and increasing frequency and severity of storm surges are anticipated to continue (Jevrejeva et al., 2016; Vermeer & Rahmstorf, 2009), as are increasing relative freshwater level rise in lakes and associated coastal shorelines and wetlands (Woolway et al., 2020). These changes are increasing the extent of coastal forest mortality (Schieder & Kirwan, 2019).

**TABLE 1:** Glossary.

Anoxia: the complete absence of O_2_.
Carbon starvation: mortality resulting from an inability to provide sufficient carbohydrate supply to metabolic processes necessary for survival.
Cytorrhysis: irreparable damage to cell walls after cellular collapse from the loss of internal positive pressure.
Die‐back: the partial loss of canopy or root biomass, without whole‐plant mortality.
Hydraulic failure: the accumulation of emboli within the sapwood past a threshold after which water transport is irrecoverable leading to dehydration and cytrorrhysis of downstream tissues.
Hypoxia: reductions in O_2_ below levels optimal for root physiology.
Mechanism: a system of parts working together within a process; a piece of the machinery.
Mortality: the irreversible cessation of metabolism and the associated inability to regenerate.
Process: a series of mechanisms that leads to an endpoint.
Salinity: the amount of salt dissolved in water, which can range from near zero in freshwater to >35 parts per thousand in seawater.

The changes in coastal ecosystem water exposure occur on a backdrop of global rises in atmospheric CO_2_, temperature, vapor pressure deficit (VPD), and drought frequency and duration (Cook et al., 2020; Friedlingstein et al., 2019; Trenberth et al., 2014; Ukkola et al., 2020), all of which lead to nonlinear interactions that challenge our understanding. An additionally large barrier to our understanding of ghost forest formation is the lack of tests of the physiological mechanisms underlying mortality, specifically conducted on dying, mature plants in coastal systems. These challenges leave our society with large predictive uncertainty regarding when and where ghosts forests will form, and their subsequent consequences on ecosystem resources (Ward et al., 2020). Improving our understanding of the drivers and processes of coastal woody plant mortality, in relation to a changing climate, is needed if we are to predict forest loss (Kirwan & Gedan, 2019).

The physiological mechanisms of hypoxia and salinity impacts on plants have been well‐studied (Colmer & Flowers, 2008; Duberstein et al., 2020; Flowers & Colmer, 2015; Flowers et al., 1977; Greenway & Munns, 1980; Krauss et al., 2009). These mechanisms include hypoxia‐ and salinity‐induced reductions in belowground hydraulic conductance, stomatal closure, reduced photosynthesis, and crown loss among others. However, these mechanisms have not yet been placed within a process framework that integrates the physiological, geomorphic, and climatic drivers and mechanisms into a predictive capacity (wherein mechanisms are components of processes; Table 1; Merriam‐Webster, 2021). Existing predictive frameworks of coastal plant loss have advanced representation of the geomorphic drivers, and in some cases, elevation‐growth relationships (Carr et al., 2020; Doyle et al., 2010; Enwright et al., 2016; Fagherazzi et al., 2019; Holmquist et al., 2021; Keddy & Campbell, 2019; Kirwan et al., 2016; Theuerkauf & Braun, 2021). Such approaches are critical to prediction because ghost forest formation depends on topographic position (Schieder & Kirwan, 2019). Integration of these frameworks with mechanistic modeling of the physiological responses to hypoxia and salinity (e.g. Yoshikai et al., 2021) could enable representation of the compounding plant responses to rising CO_2_, temperature, VPD, and droughts (McDowell et al., 2020). Integrating these disparate sets of knowledge can additionally provide a road map for future research into the formation of ghost forests under a changing environment.

Here, we bring together the mechanisms of coastal plant mortality within the framework from which the hydraulic failure and carbon starvation hypotheses originated (McDowell et al., 2008, 2022). Hydraulic failure, or the severe and irreversible loss of hydraulic conductance from xylem embolism formation that leads to dehydration of downstream tissues, appears to be a ubiquitous process in drought mortality (Adams et al., 2017): a mild degree of hydraulic failure was observed in the only study to date of the hydraulics and gas exchange patterns during coastal‐tree mortality (Zhang, Wang, et al., 2021). Hydraulic failure represents either a causal or symptomatic step in the mortality process, even in extreme experimentally induced shade or low CO_2_ conditions when mortality is driven by the carbon starvation process (Sevanto et al., 2014; Weber et al., 2018). The process of carbon starvation, or the decline in carbohydrate supply required to meet metabolic, hydraulic, and defensive needs, promotes cell rupture and mortality (McDowell et al., 2011, 2022). Severe carbon starvation was observed in the only study of the physiology of ghost forest formation in mature trees (Zhang, McDowell, et al., 2021).

The hydraulic framework is appropriate for generating hypotheses (Table 2) regarding ghost forest formation because the mechanisms underlying hypoxia‐ and salinity‐induced mortality are consistent with those included in the original framework: hydraulic and photosynthetic limitations. Even the mechanism of salt‐toxicity‐induced reductions in photosynthesis, which was not originally considered, fits squarely within the framework because of its impact on plant carbohydrate supply (Munns & Termaat, 1986). This proposed framework is consistent with the two‐phase model in which belowground osmotic changes reduce water uptake immediately upon inundation, which increases the risk of hydraulic failure and declining carbon balance, followed by a second, slower phase in which ionic impacts damage foliage, which feeds back to exacerbate carbon starvation (Munns, 1993). Hydraulic failure and carbon starvation are now simulated by ecosystem and Earth system models (e.g. Fisher et al., 2010; Jiang et al., 2016; Kennedy et al., 2019; Koven et al., 2020; McDowell, Fisher, et al., 2013); thus understanding how the individual mechanisms lead to hydraulic failure and/or carbon starvation provides a direct link to predictive modeling at multiple scales.

**TABLE 2:** Hypotheses.

Hypothesis	Description
1	Mortality is increasing globally due to rising sea levels and freshwater levels.
2	Mortality results from the interaction of topographic, hydrologic, and physiological factors.
3	Glycophytes will experience more rapid and widespread mortality than halophytes.
4	Hydraulic failure and carbon starvation are common processes, with taxa‐specific adaptations leading to a range of mortality thresholds.
5	Hypoxia and salinity drive similar mechanisms of mortality with the exception of toxicity associated with elevated salinity.
6	Hydraulic failure is driven by hypoxia‐ and salinity‐ induced reductions in belowground water uptake.
7	Carbon starvation is driven by reduced stomatal and mesophyll conductance, lost turgor, photosynthetic ion toxicity, phloem transfer reductions, crown loss, and elevated respiration.
8	Reduced growth predisposes plants to mortality through physical and physiological feedbacks.
9	Rising CO_2_ can alleviate or exacerbate mortality associated with rising water levels.
10	Rising droughts and VPD will exacerbate mortality due to rising water levels.

*Note*: Numerous hypotheses arise from application of the hydraulic framework to predict mortality from sea‐level and freshwater‐level rise. Here we list some of the most critical hypotheses that should be tested to allow improved understanding and prediction of future coastal woody‐plant mortality. These hypotheses address the interaction between topography and physiology, the physiological mechanisms underlying hydraulic failure and carbon starvation, and the potential interactions of changing CO_2_ and climate with the physiological responses to rising inundation.

Our objective is to review the state‐of‐the‐knowledge regarding coastal woody‐plant mortality in both fresh and saline environments, with the goal of enabling better informed prediction. To achieve this objective, we reviewed the literature regarding ghost forest formation from ecological, biophysical, and physiological domains to generate hypotheses regarding the underlying mechanisms of mortality for both halophytic and glycophytic woody plants. We assemble evidence from theory, observations, experiments, and models to generate a hypothesis framework. This framework is consistent with prior theories on the physiological impacts of hypoxia and salinity, while recognizing the underlying mechanisms as interdependent. This framework should also be pertinent to woody‐plants of all sizes, from regenerating seedlings to large, mature individuals, and for both halophytic and glycophytic plants. We do not focus on anthropogenic disturbances (e.g. shoreline hardening, levee construction, or deforestation) in this review; however, we cite that literature when appropriate to understand mechanisms. We do, however, briefly address anthropogenic disturbances due to their globally widespread importance and significant impacts on mortality. Likewise, while our focus is on plants located along salt‐ or fresh‐water shorelines, we do draw upon literature from upland, inland river, and wetland ecosystems where appropriate. To the degree that hypoxia drives mortality mechanisms, our framework is pertinent to both saline and freshwater inundation‐driven mortality.

## 2 PATTERNS OF WOODY‐PLANT MORTALITY IN COASTAL ECOSYSTEMS

**FIGURE 2:**
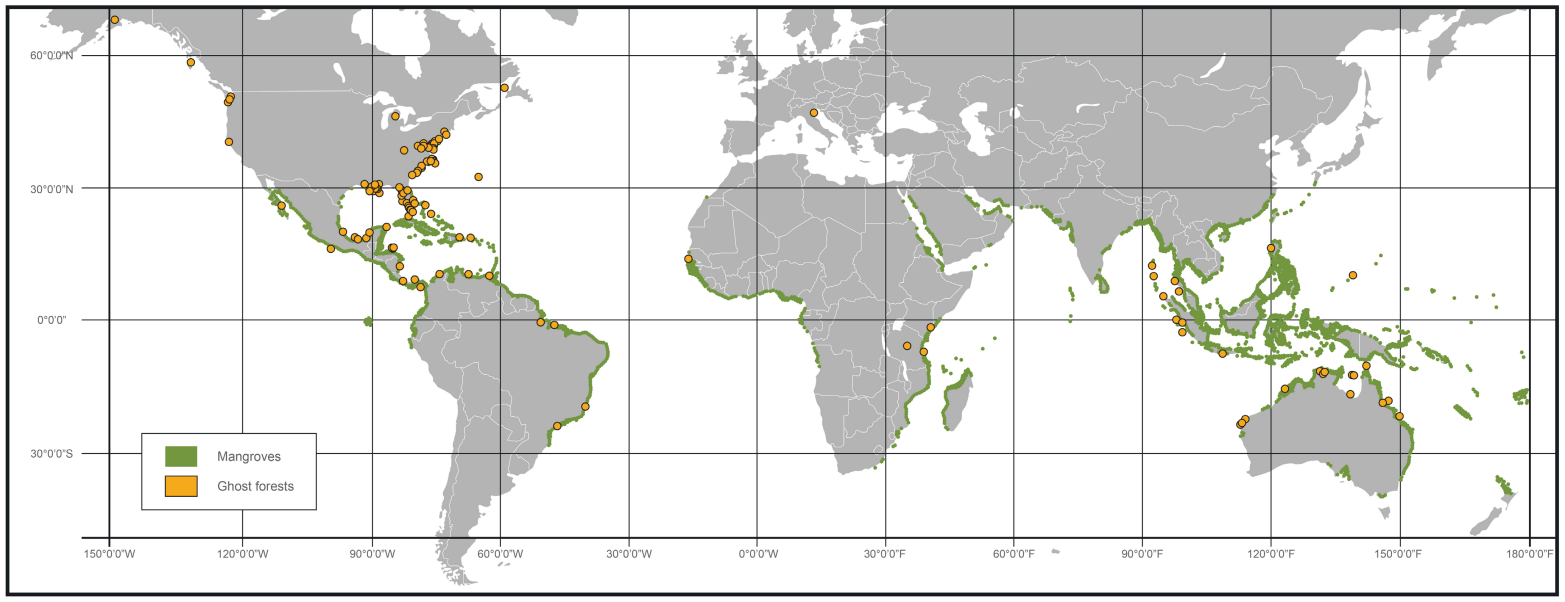
The global distribution of reported coastal mortality events resulting in ghost forests. Regions of mangrove forests are highlighted in green. Map updated with new observations, primarily from non‐mangrove systems, from Sippo et al. (2018).

The term ghost forest was coined originally to describe forest loss along the Gulf of Mexico (Penfound & Hathaway, 1938), with documented occurrences along the North American Atlantic coast dating back to 1791 (Spears, 1890; Van Doren, 1928). Ghost forests have been observed across a large range of latitudes and ecosystem types, including widespread observations in both halophytic and glycophytic species (Figure 2). Despite the variety of drivers of coastal plant mortality, the signal of climate‐ and sea level rise‐driven mortality is becoming apparent (Sippo et al., 2018). Long‐term observations are scarce, but the available site‐specific records of sufficient length show increasing rates of mortality over time (Kirwan et al., 2016; Krauss, Demopoulos, et al., 2018; Krauss, Noe, et al., 2018; Lewis et al., 2016; Schieder & Kirwan, 2019). The map in Figure 2 does not represent the global distribution of coastal mortality events in their entirety due to insufficient systematic geographical sampling. However, potential hotspots do emerge such as along the eastern coast of North America, where sea level rise combined with land subsidence, a weakening of the Gulf Stream, and relatively flat topography have promoted widespread plant mortality (Kirwan & Gedan, 2019; Sallenger et al., 2012; Schieder et al., 2018; Smith, 2013; Smith et al., 2017, 2021; Smith & Kirwan, 2021; Ury et al., 2021). The weight of this regional and global evidence leads to the hypothesis that coastal plant mortality is increasing.

Coastal woody‐plant mortality occurs in both mangrove and non‐mangrove systems (Figure 2). Cyclones, tsunamis, and hurricanes regularly cause damage and mortality in both types of systems (Lugo, 2008; Zeng et al., 2009). Likewise, mangrove mortality can occur through freeze events (Cavanaugh et al., 2019). Observations of mangrove mortality have increased in recent decades, with some individual mortality events exceeding 1000 ha along coastlines (Duke et al., 2017). These mortality events have corresponded with periods of severe heat, leading to the hypothesis that rising temperature exacerbates impacts associated with elevated hypoxia and salinity (Allen et al., 2021; Lovelock et al., 2017). This is consistent with observations and predictions from upland forests, which suggest that rising temperature, and in particular rising vapor pressure deficit (VPD), is driving increasing tree mortality (McDowell et al., 2020; Williams et al., 2013). VPD represents atmospheric evaporative demand and increases non‐linearly with temperature, causing significant impacts on plant survival through the increase in water loss relative to supply (Grossiord et al., 2020).

## 3 WOODY‐PLANT MORTALITY IN FRESHWATER SYSTEMS

Most literature on coastal woody‐plant mortality is from seashore systems, with far less information available for freshwater coastal systems, despite the critical importance of freshwater lakes and waterways for human uses (Sterner et al., 2020). Mortality of freshwater‐coastal systems has been observed in relation to shifting water levels in floodplains (Assahira et al., 2017; de Resende et al., 2019; Kramer et al., 2008; Levanic et al., 2011), and along lake shorelines (Smith, Fiorino, et al., 2021; Smith & Kirwan, 2021; Theuerkauf & Braun, 2021). Freshwater flooding typically occurs in response to anthropogenic activities (e.g. impoundments), anomalously severe precipitation events, and through climatically forced changing water levels (Gronewold et al., 2013; Kozlowski, 1984; Woolway et al., 2020). Lake levels can fluctuate at an order of magnitude faster rate than sea levels (Cazenave et al., 2014; Chen et al., 2017), forcing rapid vegetation changes along associated shorelines and wetlands (Smith, Fiorino, et al., 2021; Smith & Kirwan, 2021; Theuerkauf & Braun, 2021; Toner & Keddy, 1997).

**FIGURE 3:**
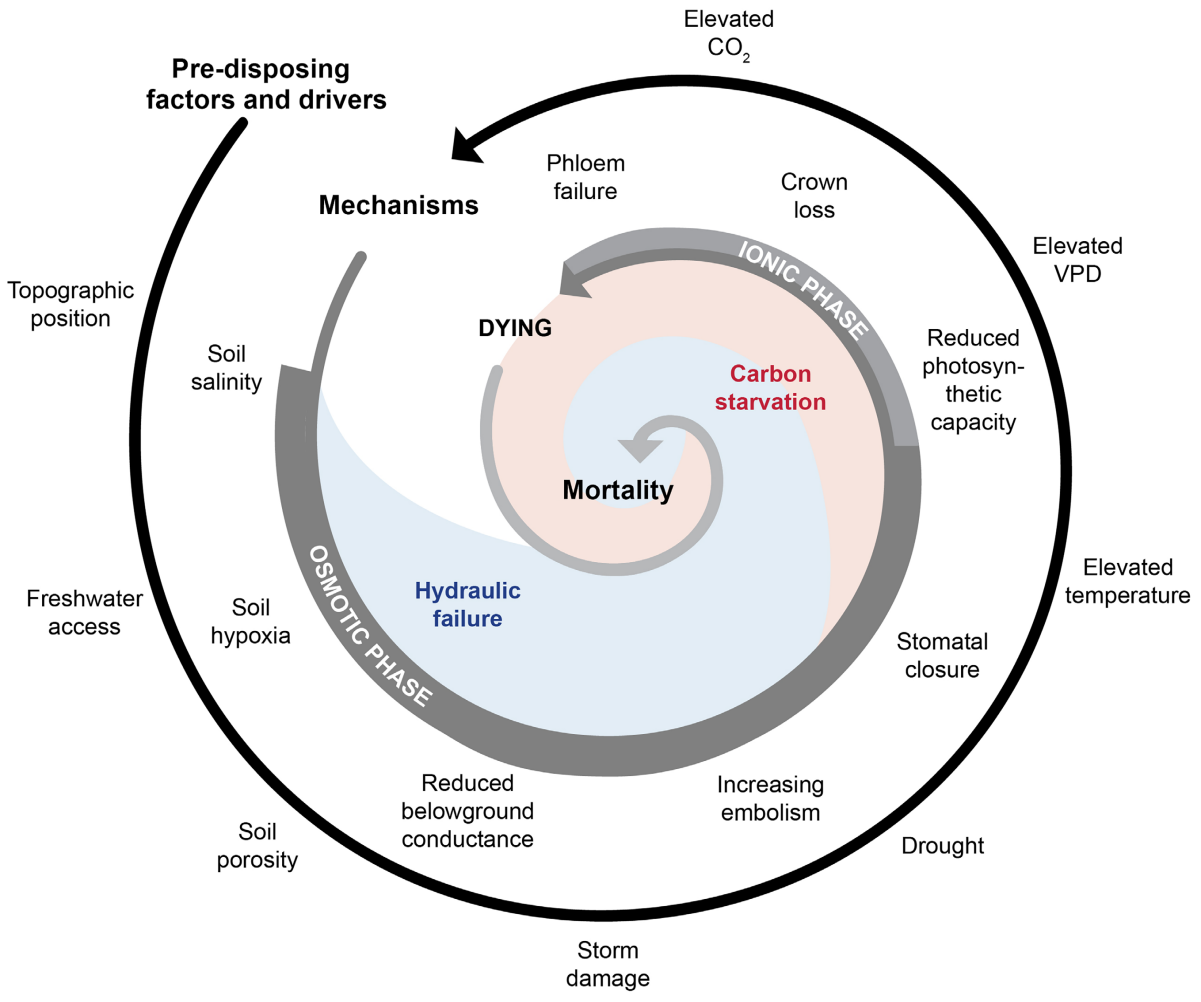
The interacting factors, drivers, mechanisms, and processes that cause coastal forest mortality under increasing seawater exposure. In this schematic, predisposing factors and drivers that occur before inundation (outer spiral) set the stage for subsequent mortality during and after inundation event(s). Pre‐disposing factors include site characteristics such as freshwater access, with drivers such as rising VPD, and mechanisms such as salt toxicity, all contributing to the processes of hydraulic failure and carbon starvation. The order of presentation of factors represents a hypothesis regarding the chronology of mechanisms and impacts. The osmotic phase (Munns & Termaat, 1986) and associated increasing risk of hydraulic failure begins as soon as saline and hypoxic conditions start upon inundation, with the ionic phase requiring more time to manifest as salts accumulate in the crown. Carbon starvation starts initially upon stomatal closure due to the associated reduction in photosynthesis. We note that increasing embolism and stomatal closure typically coincide (Rodriguez‐Dominguez & Brodribb, 2020) but are shown separately for clarity.

Freshwater and seawater systems share mortality drivers such as physical disturbance and hypoxia, and therefore share some mechanisms underlying mortality from anomalous exposure to varying water levels. Freshwater‐induced mortality is a strong function of the duration of inundation, with longer periods of inundation resulting in greater forest loss (Smith, Fiorino, et al., 2021; Smith & Kirwan, 2021). The primary driver of mortality during freshwater inundation is hypoxic conditions that prevent oxidative phosphorylation in the roots and thus create energy deficits that can result in reduced root conductance and eventual root mortality (Colmer & Flowers, 2008; Pezeshki, 2001). The subsequent reduced water uptake by roots results in stomatal closure, increased embolism, and crown loss (Figure 3). Thus, the impacts of hypoxia can lead to either or both hydraulic failure and carbon starvation, similar to mortality associated with exposure to saline water. To enable better understanding and prediction of freshwater plant mortality from inundation requires testing the subset of mechanistic responses from Figure 3 that are pertinent to hypoxia‐driven mortality. These include all the identified parameters in the framework figures except for osmotic impacts on conductance and ion toxicity to photosynthetic capacity. Ideally, studies could kill plants under varying duration of inundation with and without salinity to tease apart the relative roles of hypoxia from salt induced death, and their associated mechanisms.

We note that freshwater systems located upstream of saltwater, such as river estuaries, may experience novel salinity exposure due to rising sea level, storm surges, and human hydrologic alteration, leading to plants tolerant of hypoxia suddenly being exposed to salt (Keim et al., 2019; Stagg et al., 2016). How these differences in trait adaptations along the freshwater‐saltwater continuum confer tolerance or vulnerability to changing salinity conditions is poorly understood (Colmer & Flowers, 2008).

## 4 PREDISPOSING FACTORS UNDERLYING COASTAL WOODY‐PLANT MORTALITY

Ghost forest formation can result from the interaction of predisposing factors, external drivers, and the subsequent responses that lead to hydraulic failure, carbon starvation, and ultimately mortality (Figure 3). Predisposing factors are those that existed before the anomalous inundation event(s) that can promote either survival or mortality during and after the event(s). Site edaphic factors and external drivers provide the base from which subsequent impacts from rising water and storm surges occur. Topographic position, specifically the elevation above sea‐ or fresh‐water levels, plays a large role because it sets the potential frequency and duration of inundation (Fagherazzi et al., 2019; Schieder & Kirwan, 2019; Smith, Fiorino, et al., 2021; Smith & Kirwan, 2021; Taillie et al., 2019). In saline systems, the role of subsurface hydrology on freshwater access during and after periods of inundation plays a large role in survival (Gardner et al., 2002; Hayes et al., 2019; Semeniuk, 1983; Sternberg & Swart, 1987; Williams et al., 1999, 2003): mortality likelihood may be reduced when freshwater lenses exist within the belowground volume (soil or sediment) that is explored by plant roots, or when plants are located near rivers (Ewe et al., 2007; Guimond & Michael, 2020; Hsueh et al., 2016; Messerschmidt et al., 2021; Zhai et al., 2018). Increasing exposure to kinetic energy from elevated water levels and increasing storms can physically damage root systems (Kazemi et al., 2021) and thus reduce a plant's ability to forage for water and exchange O_2_ during the subsequent increases in hypoxia and salinity. This impact can feed back upon itself because loss of root or whole‐plant biomass to mortality will increase the kinetic energy received by the remaining surviving plants (Massel et al., 1999). Similarly, decreases in sedimentation rates, subsidence, and organic matter decomposition associated with plant mortality can feed back to produce lower soil surface elevations and hence greater exposure to inundation (Blasco et al., 1996; Cahoon, 2006). Storms, which could be increasing under climate warming (McDowell et al., 2020), can also kill plants directly through stem snapping and uprooting (Canham et al., 2010; Krauss & Osland, 2020; Lugo, 2008; Zeng et al., 2009). In surviving plants that lose a fraction of their crown, the likelihood of mortality increases significantly due in part to subsequent reductions in whole‐plant photosynthesis relative to respiring biomass, and likely subsequent carbon starvation (Gaylord et al., 2013; Poyatos et al., 2013; Zhang, McDowell, et al., 2021).

Climate and CO_2_ also play a role in predisposing plants to mortality (Figure 3). Drought (periods of anomalously low precipitation) prior to inundation can weaken trees, for example, by producing legacy embolism within the xylem and/or depleted carbohydrate stores, amplifying the likelihood of hydraulic failure and carbon starvation. Drought during or following inundation can promote mortality through the concentration of salt within pore water via high rates of evaporation with little recharge from precipitation (Ardon et al., 2013; Desantis et al., 2007). Rising temperature and VPD further exacerbate water stress through increased evaporation from both foliage and soil/sediment surfaces (Grossiord et al., 2020). Outbreaks of biotic agents such as insects or fungi can also weaken trees and thus predispose them to subsequent mortality (Gaylord et al., 2013; Werner et al., 2006). Elevated CO_2_ can either promote or mitigate the likelihood of mortality depending on the balance of increased canopy growth, which requires greater freshwater availability to avoid mortality (Duan et al., 2014), versus water savings through stomatal closure, which may promote survival by increasing freshwater availability during drought (McDowell et al., 2022). Together, each of these external drivers can predispose woody‐plants to survival or mortality under anomalous inundation (Figure 3).

Mortality during and after anomalous inundation can be lessened by vegetation traits that confer tolerance to hypoxia and salinity. Tolerance of hypoxia and hypoxia‐related toxins such as ferrous iron and hydrogen sulfide occurs across a variety of taxa and is achieved in part through aerenchyma and lenticels, higher soluble sugar contents, the ability to shift respiratory pathways, and the creation of sufficient antioxidant defenses against free‐radical generation during anaerobic conditions (Sairam et al., 2008). Halophytes, and some glycophytic woody plants adapted to wet soil conditions, can have aerial roots that increase oxygen supply belowground to mitigate hypoxia impacts (Ball, 1988).

Regarding salt tolerance, halophytes have adaptations that allow them to survive in saline environments (Acosta‐Motos et al., 2017; Zhao et al., 2020), including some species that have obligate requirements for moderate levels of pore water salinity (Nguyen et al., 2015). Halophytes have three general strategies for salt‐tolerance: osmotic adjustment enabling maintenance of turgor and favorable water relations, salt exclusion restricting salt entry into roots during water absorption, and ion compartmentation enabling safe cellular accumulation of ions away from sensitive metabolic sites (Ball, 1988). Glycophytes are more typical of upland ecosystems and have fewer adaptations to tolerate hypoxia and salinity (Greenway & Munns, 1980). For example, the presence of an exodermis within the roots is a critical adaptation because it reduces the loss of root oxygen to hypoxic soils and reduces the flux of salts into the roots (Enstone et al., 2002; Hose et al., 2001). The root exodermis is absent in all gymnosperms and common in angiosperms tolerant of drought, waterlogging and salinity (Artur & Kajala, 2021). This suggests that increasing water elevations from water level rise, storm surges, and high precipitation events in both saline and freshwater systems should have a disproportionate impact on glycophytic species without the adaptations to hypoxia and salinity, for example gymnosperms and many angiosperms. However, at some threshold, all plants become intolerant of elevated salt concentrations within the cytoplasm, with the species‐specific thresholds dependent on their adaptations (Yadav et al., 2011). This suggests convergent processes of mortality among taxa, with the mechanisms expressed at different thresholds of hypoxia and salinity, consistent with simulation results (Perri et al., 2019). Thus, we propose that hydraulic failure and/or carbon starvation could potentially manifest during inundation‐induced mortality across coastal taxa via shared mechanisms, with edaphic, predisposing, and vegetation traits producing effects at varying levels of hypoxia and salinity.

## 5 MECHANISMS UNDERLYING MORTALITY FROM HYPOXIA AND SALINITY

**FIGURE 4:**
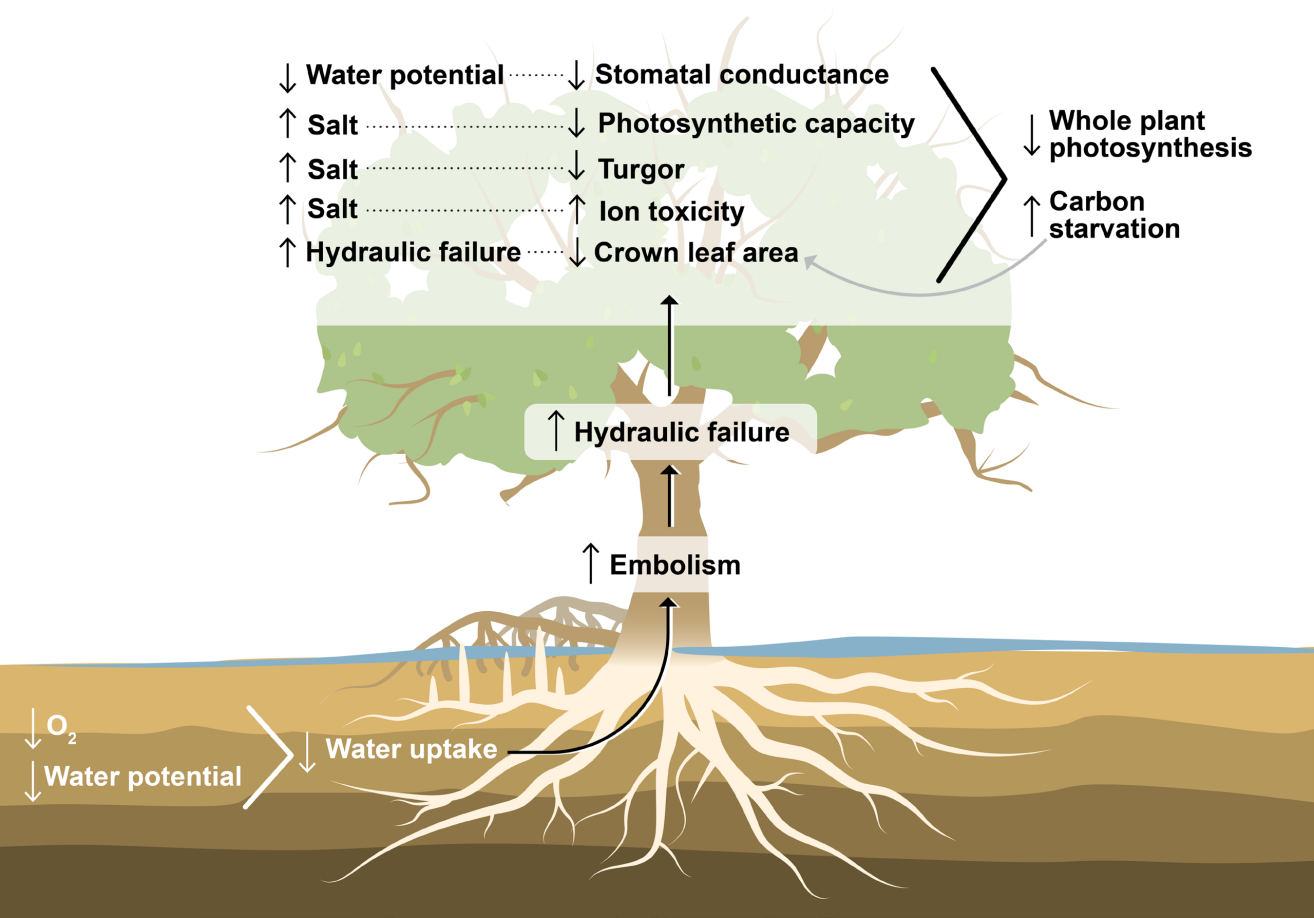
A mechanistic framework for coastal forest mortality from hypoxia and salinity. Abnormally prolonged or repeated exposure to hypoxia or high salinity results in significant reductions in belowground hydraulic conductance due to root death and osmotically induced reductions in the water potential gradient from soil to root. Reduced belowground conductance results in declining water supply to the foliated crown, increasing embolism and subsequently hydraulic failure. Reductions in water flow to the crown simultaneously induce carbon starvation through multiple mechanisms. Decreased foliar water potential reduces stomatal conductance while increasing foliar salt concentrations cause reduced photosynthetic capacity, turgor, and direct ion toxicity to cellular structure, all leading to decreased photosynthesis and hence increased risk of carbon starvation. Carbon starvation feeds back on itself through mortality of foliage via a negative carbon balance, leading to crown dieback and hence reduced whole‐plant photosynthetic capacity. Ultimately, the combination of these factors increases the likelihood of both hydraulic failure and carbon starvation.

Understanding and predicting mortality under future increases in water inundation, atmospheric CO_2_, and VPD requires a framework that provides unambiguous definitions, generates testable hypotheses, and identifies uncertainties. Such a framework should also identify pools and fluxes of critical resources and their potential interdependencies and lethal thresholds, and should be relevant across different taxa and environmental conditions. Here, we propose that the hydraulic framework, from which the hydraulic failure and carbon starvation hypothesis originated, satisfies these criteria, enabling prediction of coastal woody‐plant mortality when integrated with existing knowledge regarding inundation impacts. For seashore plants specifically, we integrate the hydraulic framework with the proposed two‐phase osmotic and ionic impacts framework, in which water limitations drive rapid, widespread reductions in photosynthesis, which compound with the slower but equally damaging impacts of ion toxicity on photosynthesis and leaf mortality (Munns, 1993). We propose that hydraulic failure and carbon starvation drive the final step in mortality, widespread cellular rupture (cytorrhisis; Beckett, 1997; Ding et al., 2014), through the progressive impacts of reduced O_2_ and pore water osmotic potential and subsequent constraints on root water uptake. Declining water acquisition promotes an increase in the risk of hydraulic failure while photosynthetic limitations due to reduced stomatal conductance, salt toxicity, and crown loss simultaneously promote carbon starvation (Figures 3 and 4). Notably, these are all whole‐plant responses because, even if they start at the organ level, they eventually manifest throughout the plant, for example individual root death or branch‐level hydraulic failure. Because anomalous inundation starts by reducing water transport capacity and subsequently leads to impacts on carbon balance, we first review the response of plant water relations to inundation and salinity exposure, with subsequent emphasis on carbon relations.

### 5.1 Hydraulic failure

Mortality of seawater‐exposed woody plants begins belowground with prolonged exposure to reduced O_2_ and in seawater shorelines, increasingly saline pore water (Figure 3). Both hypoxia and elevated salinity reduce the water uptake capacity of roots (Drew, 1997; Duberstein et al., 2020; Muller et al., 2009; Nicolás et al., 2005; Krauss & Duberstein, 2010; Krauss et al., 2014; Pedersen et al., 2021), forcing a large decline in transpiration that subsequently triggers the mechanisms underlying hydraulic failure and carbon starvation (Figure 4). Hypoxia impacts the roots' capacity to acquire water due to reduced hydraulic conductance and ultimately through root death from O_2_ starvation of respiration and toxic impacts on root metabolism (Dat et al., 2006; Malik et al., 2002; Pedersen et al., 2021; Sairam et al., 2008; Youssef & Saenger, 1998). This loss of belowground water uptake decreases water supply relative to the whole‐plant demand and hence places increased tension within the hydraulic system, elevating the risk of runaway cavitation within the xylem, leading to loss of water transport to downstream tissues, that is, hydraulic failure (Tyree & Sperry, 1989).

Even relatively small increases in salinity, when they occur in ecosystems adapted to freshwater dominance, can lead to rapid ghost forest formation (Pezeshki et al., 1987; Salinas et al., 1986). High salt concentrations can linger or even increase following the flooding event depending on subsequent freshwater input, VPD (through its impact on evaporation), soil hydraulic conductivity, and cation exchange capacity (Ardon et al., 2013; Fagherazzi et al., 2019). Rising salinity of soil pore water induces a cascade of changes in plant water relations driven foremost by the elevated osmotic potential of saline water, which subsequently reduces the potential gradient for water flux through the rhizosphere (Figure 4; Boursiac et al., 2005). Standard seawater has an osmotic potential of −2.4 MPa (Harvey, 1966), which is lower than root water potential for most coastal taxa, and thus exposure to saline pore water can reduce or eliminate water flow into roots. Additionally, root hydraulic conductivity declines with increasing salinity, exacerbating the decline in root water impact beyond that of the osmotic impact (López‐Berenguer et al., 2006; Nedjimi, 2014). This is called the osmotic phase of plant response to salinity (Figure 3; Munns & Tester, 2008). These mechanisms lead to reduced water uptake that promotes reductions in leaf water potential and subsequently declines in stomatal conductance, which minimizes transpiration and thus reduces the risk of hydraulic failure (Clough & Simm, 1989; Orsini et al., 2012; Perri et al., 2019; Sperry et al., 2016). Stomatal closure also aids in avoiding turgor loss (Méndez‐Alonzo et al., 2016). However, under these conditions hydraulic failure becomes increasingly likely as water loss continues through the cuticle and bark even after complete stomatal closure (Cochard et al., 2021; Wolfe, 2020), resulting in a progressively more negative water balance (Figure 4). Stomatal closure also has implications for carbon starvation through reduced photosynthesis via reduced CO_2_ diffusion through stomata (Figures 3 and 4).

Hydraulic failure from salinity or hypoxia‐driven root death can lead to partial or complete crown mortality, and to mortality of entire individuals if it occurs widely throughout the entire plant, including the meristems that are the source of regenerating tissue. Hydraulic failure in dying coastal plants has not been examined in depth, but recent evidence from conifer trees exposed to novel tidal inputs of saline water demonstrated that mortality was associated with up to 60% loss of xylem conductance (Zhang, Wang, et al., 2021). Increased resistance to hydraulic failure caused by exposure to saline and hypoxic conditions, for example acclimation may occur in seedlings (Pandolfi et al., 2012, 2016; Pezeshki et al., 1987; Stiller, 2009), but this has not been investigated in mature plants to our knowledge.

Declining foliar turgor due to increasing salinity exposure can promote hydraulic failure by driving plants towards their lethal threshold for runaway cavitation and formation of widespread xylem emboli (Bartlett et al., 2016; Méndez‐Alonzo et al., 2016; Reef & Lovelock, 2015; Figure 4). Additionally, reductions in turgor associated with higher salinity cause reduced cell expansion during growth, leading to fewer and/or smaller conduits for water to move through the xylem (Munns, 1993). These smaller elements subsequently reduce hydraulic conductance, and without reductions of distal foliar biomass, the rate of water supply‐to‐demand decreases significantly, increasing the tension within the xylem. Thus, sustained turgor loss anywhere in the root‐to‐leaf hydraulic system may cascade towards an accumulation of xylem emboli and lead to hydraulic failure.

### 5.2 Carbon starvation

**FIGURE 5:**
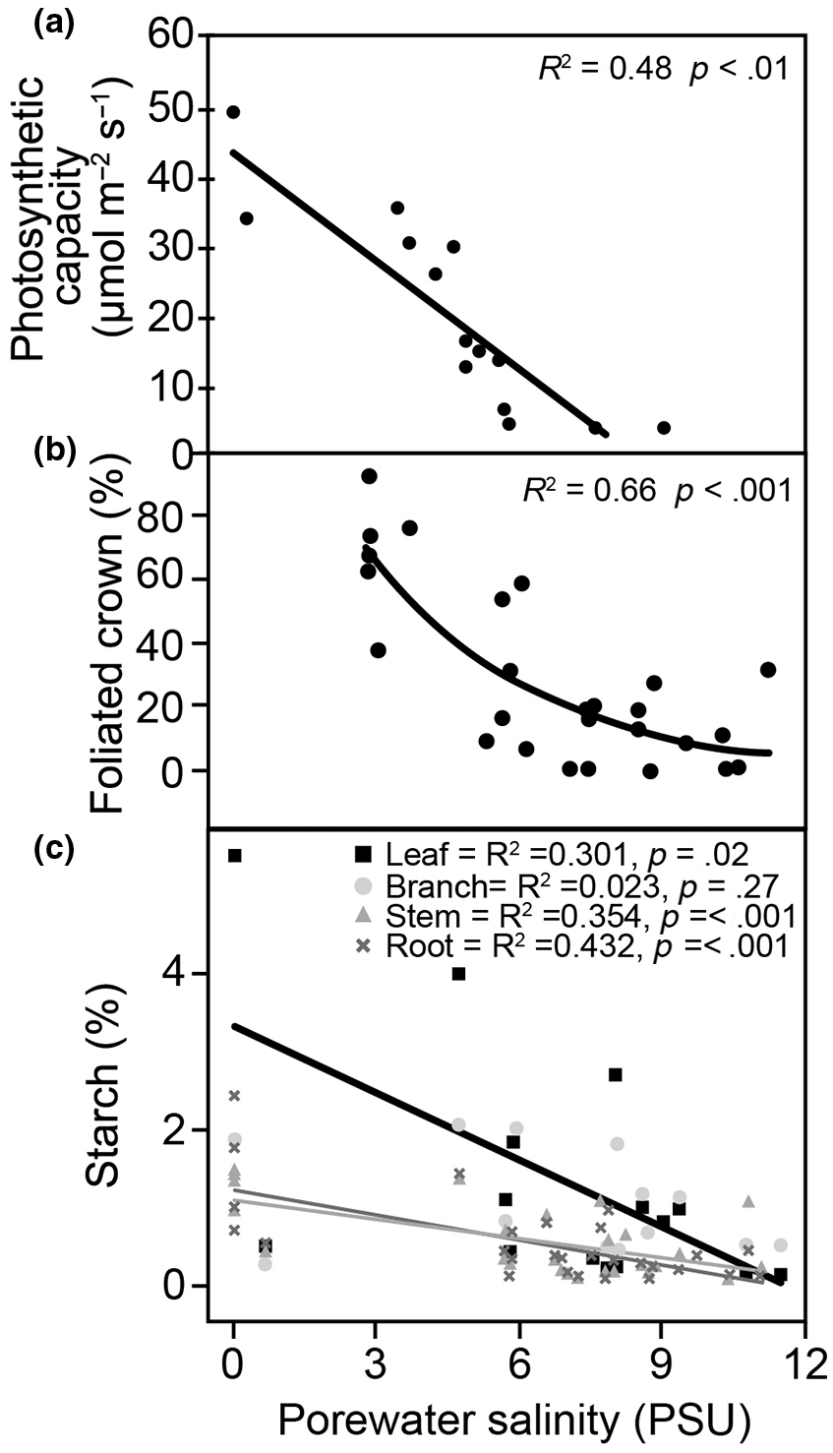
Declining tree carbon uptake promotes carbon starvation. Sitka spruce (*Picea Sitchensis*) trees exposed to seawater inundation exhibit strong declines in (a) photosynthetic capacity (the slope of photosynthesis versus internal CO_2_ concentration), (b) the fraction of their crowns that are foliated, and (c) starch concentrations in the foliage, branches, stems and roots. Combined, the decline in both photosynthetic capacity and in crown leaf area result in a significant decline in starch concentrations, which is the primary storage pool of carbon. As trees approach death the starch concentrations reach zero, which is rarely seen except for in studies that force carbon starvation (Quirk et al., 2013; Sevanto et al., 2014; Weber et al., 2018). Data used to create these figures are from Li et al. (2021) and Zhang, McDowell, et al. (2021).

Carbon starvation is the process by which limitations of carbon supply relative to demand cause failure to maintain metabolic and hydraulic functions above thresholds that prevent widespread cellular cytorrhysis (McDowell et al., 2022). Inundation increases the likelihood of starvation through reducing the ratio of carbon uptake to demand that manifest at leaf‐ and whole‐plant scales over minutes to years (Figures 3 and 4). The first impacts of increased hypoxia and salinity occur by the aforementioned reduction in belowground hydraulic conductance (Figure 4). Reductions in hydraulic conductance force declines in CO_2_ diffusion into foliage, and hence photosynthesis, due to stomatal closure (Figure 5a; Ball & Farquhar, 1984; Brugnoli & Lauteri, 1991; Li et al., 2021; Mustroph & Albrecht, 2003; Zhang, Wang, et al., 2021). This reduction in whole‐plant photosynthesis is the most immediate mechanism driving carbon starvation (Wang et al., 2019). A second mechanism of reduced photosynthesis is declining mesophyll conductance to CO_2_ induced by elevated salinity (Flexas et al., 2004). A third mechanism of reduced photosynthesis is turgor loss resulting from salinity stress, because photosynthetic rates decline with declining turgor and reach zero photosynthesis when turgor is lost (Figure 4; Brodribb et al., 2003; Méndez‐Alonzo et al., 2016).

A fourth mechanism driving a reduction in whole‐plant photosynthesis under seawater exposure occurs via salt‐induced damage to photosynthetic biochemistry (Figures 3, 4, 5; Ball & Farquhar, 1984; Delatorre‐Herrera et al., 2021; Li et al., 2021; Yadav et al., 2011). Salt toxicity takes longer to manifest than the more immediate hypoxic and osmotic limitations upon water uptake (Munns, 1993) due to the time lag in salt transport to shoots via the transpiration stream and the time required for salt to accumulate to toxic levels. Accumulation of salts in the foliar cytoplasm, and inhibition of nitrogen uptake, both directly impact photosynthetic capacity by reducing electron transport and carboxylation capacities (Ball et al., 1987; Ge et al., 2014; James et al., 2006; Munns, 2005; Suárez & Medina, 2006). Salt accumulation in foliage can thus lead to dramatic reductions in photosynthetic capacity, particularly in glycophytes that have limited ability to exclude salts at the root surface (Figure 5a; Li et al., 2021).

A fifth mechanism that reduces carbon uptake at the whole‐plant scale is crown dieback, or foliage loss (Figure 4; Munns & Termaat, 1986), which is commonly observed during the formation of ghost forests (Lovelock et al., 2017; Peters et al., 2021; Zhang, Wang, et al., 2021). Crown loss is driven by both hydraulic failure through severe reductions in water supply leading to cellular cytorrhysis and carbon starvation through the above‐mentioned mechanisms of reduced photosynthesis that drive a net negative carbon balance, again leading to cytorrhysis (McDowell et al., 2022). Additionally, if salt concentrations reach sufficiently high levels, programmed cell death occurs leading directly to cytorrhysis (Joseph & Jini, 2010; Katsuhara & Shibasaka, 2000; Li et al., 2007). Critically, crown loss from carbon starvation promotes further crown loss due to the loss of whole‐plant photosynthesis, leading to a feedback spiral of negative carbon balance (Figures 3 and 4). Such reductions in whole‐plant foliar surface area are sometimes considered a mode of acclimation to salinity stress because they reduce water demand (Acosta‐Motos et al., 2017; Rodrıguez et al., 2005); however, we argue that beyond a certain threshold, crown loss is a causal mechanism in mortality, as this loss of whole‐plant photosynthesis further limits the plants' capacity to store carbohydrates and to grow foliage, xylem, and roots (Figures 4 and 5b; Greenway & Munns, 1980; Li et al., 2021; Munns & Termaat, 1986). Ultimately, these five limitations on whole‐plant photosynthesis from hypoxia and salinity drive declines in carbohydrate storage that promotes carbon starvation (Figure 5c; Zhang, McDowell, et al., 2021), which is consistent with the prediction of feedbacks between the osmotic and ionic drivers upon reduced carbohydrate storage with decreasing crown size (Munns & Termaat, 1986).

Beyond reduced photosynthesis, the process of carbon starvation can be exacerbated if there is an insufficient transport rate of carbon substrates to required metabolic processes (Yu, 1999). Increased risk of carbon starvation can result from transport constraints through source and sink strength impacts upon phloem loading and unloading, and increasing viscosity (Figure 4; Mencuccini et al., 2015; Saglio, 1985). The impact of salinity upon phloem function is poorly understood in seashore plants, but there is reason to suspect that phloem flow can be reduced under highly saline conditions (Perri et al., 2019). Lowered turgor due to increasing salt concentrations within the foliage apoplast (e.g., Ottow et al., 2005; Zeng et al., 2009) can reduce phloem water transport, especially when associated with reduced carbohydrate availability to drive water flow, as occurs with inundation (Chen & Polle, 2009). Reduced xylem water potentials due to constrained water uptake will reduce phloem water content, concentrating phloem sucrose and elevating the viscosity and hence further reducing flow (Hölttä et al., 2009; Jensen et al., 2013). High osmotic pressure in the roots, if not accompanied by effective salt extrusion processes, can reduce phloem sink strength, potentially eliminating flow of carbohydrates (Abdolzadeh et al., 2008; Matsushita et al., 1992; Yang et al., 2019). Notably, osmoregulation could counteract reduced phloem transport if it generates sufficiently elevated osmolyte concentrations to maintain flow (Perri et al., 2019; Plaut et al., 2012). Thus, salinity and hypoxia can exacerbate localized carbon starvation through limitations to phloem flow, with the threshold for carbon starvation varying with adaptations to maintain phloem function. While these hypotheses are relatively well supported with evidence from studies of surviving plants in upland systems (Chen & Polle, 2009; Wu, 2018), they should be tested on dying plants in coastal systems in order to draw robust conclusions (McDowell, Ryan, et al., 2013).

Concurrently with decreasing carbon supply due to hypoxia‐ and salinity‐exposure, the metabolic demand for carbon could increase as plants shift resource allocation to promote survival. Carbohydrates are needed in part for regulation of xylem sap osmotic potential, which contributes to the soil‐to‐root water potential gradient (Acosta‐Motos et al., 2017). Declining resistance to xylem embolism has been associated with low carbohydrate concentrations in multiple experimental and observational studies, suggestive that reduced carbon balance can promote a hydraulic‐carbon feedback (Tomasella et al., 2020). Respiratory consumption of carbohydrates increases for many reasons under hypoxia and salt stress (Jacoby et al., 2011, 2013). Carbohydrates are consumed for use in both cellular osmoregulation and for free‐radical scavenging, the requirements of which increase under salt‐stress (Acosta‐Motos et al., 2017; Jouve et al., 2004; Munns & Gilliham, 2015). The demands to maintain protein and membrane stability and cross‐membrane transport of salts and amino acids increase under hypoxia and salt stress (Hoekstra et al., 2001; Koster & Leopold, 1988; Matros et al., 2015; Ramel et al., 2009; Rolland et al., 2006; Sapes et al., 2021; Van den Ende & Valluru, 2009; Yu, 1999), thus creating feedback loops that promote subsequent starvation. Finally, increased respiration occurs through shifts in metabolic pathways used to maintain metabolism under hypoxia (Drew, 1997) and under salt stress (Che‐Othman et al., 2020). All of these increases in carbon demands suggest that the risk of localized or widespread decreases in photosynthesis, coupled with simultaneous increases in carbohydrate demands (e.g. Figure 5a,b), can increase the likelihood of carbohydrate levels falling below thresholds for survival (e.g. Figure 5c).

A final driver of mortality related to plant carbon relations under hypoxia and salinity stress is reduced growth (Ball, 1988). Reduced root and mycorrhizal growth limit the ability of plants to forage for freshwater (Evelin et al., 2009; Hsueh et al., 2016; Sternberg & Swart, 1987; Zhai et al., 2018). Because roots play a critical role in sediment accretion (Kirwan & Megonigal, 2013; Kumara et al., 2010), reduced root growth could cause decreased rates of sedimentation, leading to greater subsequent submergence (Cahoon, 2003; Krauss, Demopoulos, et al., 2018; Krauss, Noe, et al., 2018). Reduced carbon allocation to suberin in the roots, which excludes salt, can promote greater salt uptake (Reef & Lovelock, 2015). Xylem growth dominates recovery of hydraulic conductance after embolism at mid‐ to long‐term scales (Brodribb et al., 2010), hence lower xylem growth means less recovery of hydraulic conductance and greater risk of both hydraulic failure and carbon starvation. Finally, reduced growth of foliage limits whole‐plant photosynthetic potential, thereby promoting subsequent starvation (Poyatos et al., 2013). Together, these growth reductions drive hydraulic, carbohydrate, and sedimentation impacts that promote hydraulic failure and carbon starvation.

The bulk of evidence points to initial hydraulic limitations of hypoxia and salinity driving a cascade of impacts that can lead to hydraulic failure and carbon starvation, particularly when coupled with ion‐toxicity to photosynthesis and reduced crown surface area (Figures 3, 4, 5). The manifestation of these mechanisms may vary under rising CO_2_ and changing climate, thus we next consider the hypothesis framework under these increasingly important drivers of plant physiology.

## 6 THE ROLE OF RISING CO_2_ , DROUGHT, AND VPD ON COASTAL WOODY‐PLANT MORTALITY

**FIGURE 6:**
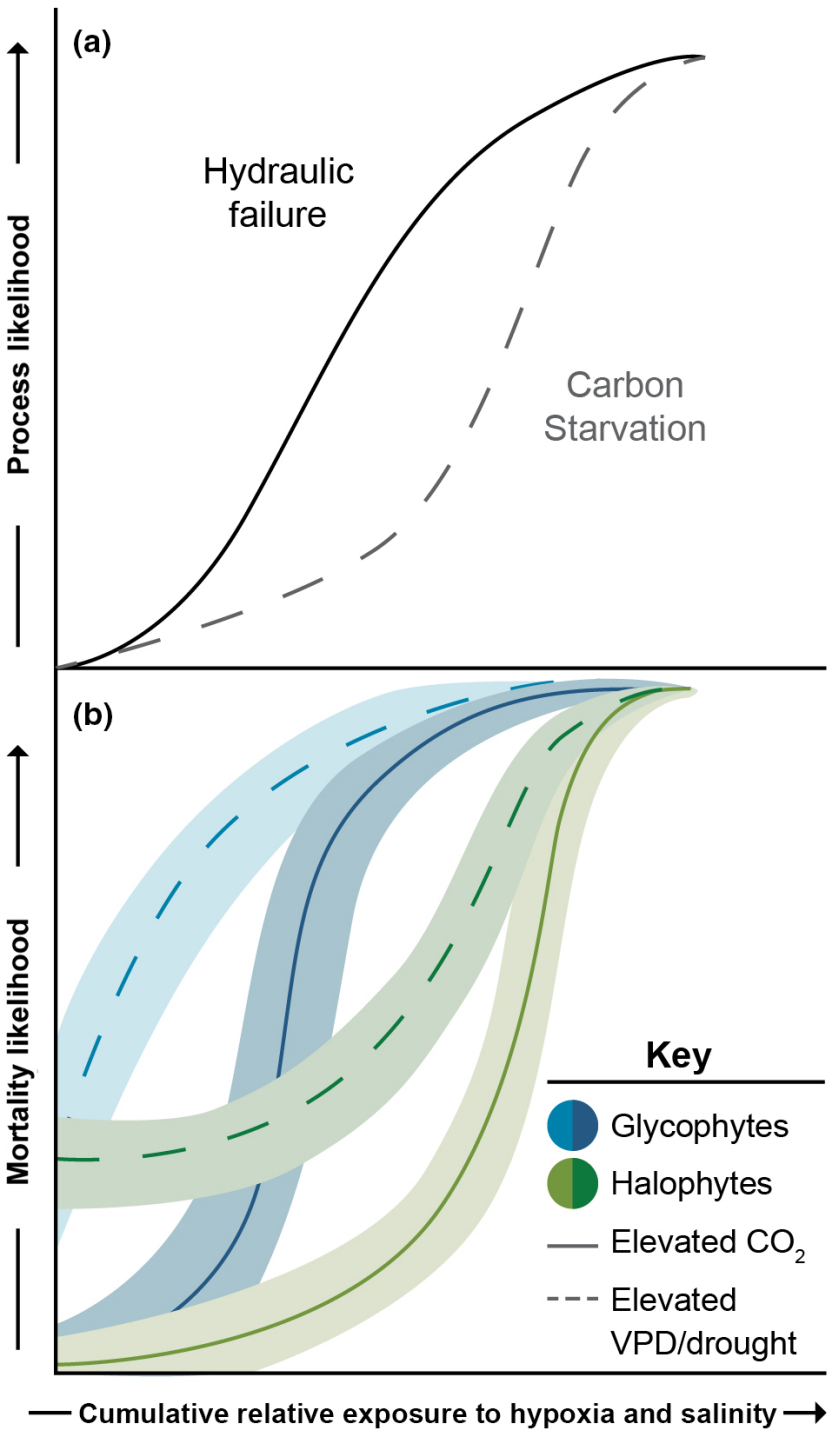
Hypothesized responses of mortality to rising hypoxia and salinity and the influence of rising CO_2_, VPD, and droughts. (a) As the cumulative duration of exposure to elevated hypoxia and salinity increases, hydraulic failure should be the first process to manifest because of the immediate declines in belowground hydraulic conductance that subsequently promote increased embolism, crown dieback, and eventual mortality. Carbon starvation will increase in likelihood at a slower rate because of the buffering capacity of stored carbohydrates but can become a dominant driver of mortality due to the large declines in whole‐plant photosynthetic potential. (b) Glycophytes are more sensitive to hypoxia and salinity than halophytes, however, their responses to elevated CO_2_ and VPD are similar. Elevated CO_2_ can mitigate mortality likelihood at low levels of hypoxia and salinity, but these benefits are lost with rising hypoxia and salinity. The relative range of exposure shown in panel (b) allows for a range of exposure at across taxa, topography, soil depths, and climate among other variables that could drive variation in any convergence. Elevated VPD, in contrast, will increase mortality likelihood throughout the range of hypoxia and salinity, regardless of vegetation‐type.

Atmospheric CO_2_, droughts, and VPD are all rising rapidly and are expected to continue increasing for the foreseeable future (IPCC, 2021), but the interactions of these drivers with hypoxia and salinity in causing mortality are largely unknown. We hypothesize that while halophytes and glycophytes have different sensitivities to hypoxia and salinity, they have similar responses to rising CO_2_, VPD, and drought (Figure 6). This convergence hypothesis results from the consistent evidence suggesting that halophytes and glycophytes may both die via the same mechanisms, just at different points on the hypoxia and salinity curves (Figure 6; reviewed above). Below, we review the specific mechanisms underlying potential shifts in mortality due to rising CO_2_, VPD, and drought.

In mesic, young, upland ecosystems, CO_2_ fertilization can decrease stomatal conductance (Ainsworth & Rogers, 2007; Klein & Ramon, 2019), and can increase total biomass (Walker et al., 2021), fine root biomass (Anderson‐Teixeira et al., 2013), rooting depths (Iversen, 2010), plant leaf area (Norby & Zak, 2011; Walker et al., 2021), and leaf biomass to total biomass ratio (Lovelock et al., 2016). Specific to seashore systems, CO_2_ fertilization can reduce oxidative stress under elevated salinity (Geissler et al., 2009). CO_2_ fertilization can reduce the likelihood of mortality (Figure 6) if carbohydrate storage is enhanced by CO_2_ (Li et al., 2018). Water savings from reduced stomatal conductance could maintain more freshwater availability to plants (Keenan et al., 2013; Niklaus et al., 1998; Walker et al., 2021).

Not all CO_2_‐induced changes are beneficial to survival (McDowell et al., 2022). The increased demand for water due to elevated growth of the plant canopy could nullify any stomatal water savings by increasing whole plant water demand during stressful periods (Duan et al., 2014). This concept is referred to as ‘structural overshoot’, where plants cannot provide sufficient water to supply the larger crown during periods of resource limitation (Jump et al., 2017; Ward et al., 2013, 2015; Tor‐ngern et al., 2015). This whole‐plant response may explain why nutrient enrichment promotes mangrove mortality (Lovelock et al., 2009), given that elevated nutrient availability typically increases the ratio of above‐ to below‐ground biomass and thus predisposes trees to mortality under drought due to elevated water demand relative to supply (Ewers et al., 2001). Consistently, while CO_2_ benefits plant growth under relatively low salinity, it does not benefit plants under elevated salinity levels (Figure 6; Alongi, 2015; Ball et al., 1987). This is likely due to limited stomatal conductance to CO_2_ via osmotic constraints on water flow and due to exceedance of the threshold for photosynthetic damage due to salt toxicity, both of which can nullify the benefits of elevated CO_2_ (Reef et al., 2016). This leads to the hypothesis that while CO_2_ fertilization may or may not reduce mortality likelihood at low levels of hypoxia and salinity, the likely response to increasingly low O_2_ and high salinity conditions should be a particularly steep response of mortality likelihood due to structural overshoot (Figure 6). Thus, chronic increases in inundation events should overwhelm CO_2_ benefits in locations where water level rise is accelerating.

Meteorological droughts, when precipitation is anomalously low, are anticipated to become more frequent (Ukkola et al., 2020) and can drive widespread mortality in coastal systems (Figure 6; Desantis et al., 2007; Lovelock et al., 2017; Sippo et al., 2018). Drought prior to extreme inundation events can predispose plants to mortality through increased xylem embolism and reduced carbohydrate storage and growth, thus exacerbating the likelihood of hydraulic failure and carbon starvation. Similarly, drought during or after inundation should promote these mortality processes through exacerbating the negative impacts of hypoxia and salinity on embolism likelihood, and subsequent reductions in photosynthesis and increased likelihood of mortality (Figures 3 and 4). Drought after seawater inundation will increase soil or sediment pore water salinity (Lovelock et al., 2017), thus exacerbating osmotic impacts. Drought combined with inundation events are thus compounding drivers of hydraulic impacts that should exacerbate the risk of hydraulic failure and carbon starvation (Figure 6).

VPD is rising in concert with increasing atmospheric temperature (Grossiord et al., 2020), with the increase in soil and leaf evaporation increasing mortality likelihood under anomalous water inundation (Figure 6). Prior to stomatal closure, VPD‐induced increases in transpiration elevate the risk of hydraulic failure as water demand becomes larger than supply, particularly when hydraulic supply is reduced due to hypoxia and salinity. After stomatal closure, elevated VPD continues to drive water loss through the foliar cuticle and bark, which becomes the dominant source of water loss after stomatal closure (Duursma et al., 2019). This residual, non‐stomatal water loss exhibits a strongly non‐linear, positive response to rising temperature, and heat waves are thus of particular concern for accelerating water loss and the likelihood of hydraulic failure (Duursma et al., 2019).

In seashore systems exposed to significant tidal fluctuations, increased evaporation due to rising VPD can cause elevated soil pore water salinity through concentration of salts during low‐tides (Cormier et al., 2013; Desantis et al., 2007). This process of increasing salinity could have played a large role in the recent, widespread mangrove mortality in northern Australia, in which a drought/high VPD period corresponded with a period of particularly low sea level that resulted in extremely high pore water salinity concurrent with mortality (Lovelock et al., 2017). Thus, we hypothesize that the mortality likelihood is higher across the possible range of hypoxia and salinity under elevated VPD (Figure 6). Together, rising CO_2_, VPD, and drought are likely to push plants towards accelerated mortality under rising hypoxia and salinity, though some degree of stress alleviation through elevated CO_2_ might occur at low degrees of hypoxia and salinity (Figure 6).

## 7 ANTHROPOGENIC DISTURBANCES

Human‐induced disturbances along coastal shorelines are globally widespread (Goldberg et al., 2020; Kirwan & Gedan, 2019). Such disturbances can have significant, large‐scale impacts on coastal woody‐plants via the same mechanistic responses as to increases hypoxia and salinity (Figures 3 and 4). These disturbances are often in the form of deltaic system alteration such as river dredging, and construction of dams, levees, navigation canals, road construction, and seawalls (Kirwan & Gedan, 2019; Lewis et al., 2016; Salinas et al., 1986). In many regions globally, conversion of land to aquaculture or agriculture is a dominant disturbance (Goldberg et al., 2020) as is tree cutting for timber (Romañach et al., 2018). Such landscape alterations can result in large‐scale mortality due to shifts in hydrologic and sediment fluxes (Jones et al., 2017; Syvitski et al., 2009). Drying or ponding of freshwater dominated ecosystems due to hydrologic shifts can promote mortality through severe reductions in soil moisture availability or increased hypoxia, respectively. If such shifts result in increased intrusion of saline water, then osmotic and ionic impacts increase. Loss of woody‐plants due to human disturbance can result in reduced rates of sediment accumulation and compaction (Lang'at et al., 2014), while planting increases surface elevation through increased sedimentation. These changes in elevation make large differences in plant exposure to hypoxia, salinity, and kinetic energy from waves. Fragmentation and reduced stem densities likewise increase mortality from storm surges (Kumara et al., 2010; Lagomasino et al., 2021).

Protection of coastal ecosystems dominated by woody‐plants is a large challenge (Lee et al., 2019; Osland et al., 2018). Conservation is complex due to the wide range of land stewardship of shorelines globally (Titus et al., 2009) and because of the required coordination between national policies and local communities (Romañach et al., 2018). Avoiding the construction of shoreline structures through coastal land conservation is critical, while restoration of degraded systems can require investment into structures that move the local hydrology back towards a pre‐disturbance state (Temmerman & Kirwan, 2015). Economic factors play a significant role influencing restoration due to construction costs (Hinkel et al., 2014). Balancing flood mitigation with restoration is another critical factor determining the ability to restore damaged ecosystems (Calil et al., 2015). Global conservation agreements have resulted in protection of large swaths of coastal forests, as have public outreach efforts to educate society regarding the ecosystem services lost through coastal plant mortality (Romañach et al., 2018). Ultimately, balancing the economic benefits of coastal development against the ecosystem services provided by intact ecosystems can drive policy‐ and decision‐making regarding future human‐induced disturbances (Barbier et al., 2011; Friess et al., 2016; Hochard et al., 2019).

## 8 FUTURE DIRECTIONS

There have been significant advances in our understanding of how hypoxia and salinity impact plant physiology (Ball & Farquhar, 1984; Kozlowski, 1997; Longstreth & Nobel, 1979; Munns, 1993; Munns & Gilliham, 2015). The various deleterious impacts of hypoxia and salinity on root function, water uptake and demand, photosynthetic biochemistry, carbon transport and metabolism, and on turgor are relatively well understood at cellular and molecular levels (Gupta & Huang, 2014; Hasegawa et al., 2000; Munns & Tester, 2008). However, the application of this mechanistic knowledge to understanding coastal woody‐plant mortality from hypoxia and salinity exposure is relatively unexplored.

We suggest a testable framework for understanding and modeling inundation‐ and salinity‐induced mortality (Figures 3 and 4). We hypothesize that hypoxia‐ and salinity‐driven shifts in water uptake increase the risk of hydraulic failure and carbon starvation through multiple mechanisms (Table 2). The immediate and sustained reductions in hydraulic conductance due to belowground osmotic constraints and potentially root death should simultaneously increase likelihoods of hydraulic failure and carbon starvation, with interdependent feedbacks between them (Figure 4). Photosynthetic declines due to declining stomatal conductance, ion toxicity to photosynthetic biochemistry, reduced phloem transport, and loss of crown surface area, combined with increasing respiratory demand, all should drive large reductions in carbohydrate supply (Figure 5). We additionally hypothesize that rising CO_2_ can either benefit or hurt plants during inundation events, whereas rising VPD will predispose plants to mortality above that expected from inundation alone (Table 2 and Figure 6). These and other hypotheses embodied by the framework should be testable in both field and numerical modeling experiments.

This mechanistic framework allows identification of both challenges and solutions to understanding and modeling coastal woody‐plant mortality. Critically, there remain too few observations of the global distribution of ghost forests to allow strong conclusions about geographic patterns or trends of ghost forest formation over time (Figure 2). There is also a paucity of measurements on mature coastal plants needed for testing the hypotheses proposed within Figure 4. A major need is increased data generation from coastal, dying woody plants that targets the key mechanisms (Figure 4). At a minimum, both changes in hydraulic conductance and carbohydrate concentrations should be quantified in dying and surviving plants for testing of the hydraulic failure and carbon starvation hypotheses (Figures 3 and 4). Identifying when and where belowground conductance approaches zero due to osmotic constraints is critical because this sets the remainder of the mortality mechanisms into motion (Körner, 2019). Likewise, identifying dehydration and carbon supply thresholds and how long plants can survive on water stores, while losing water to residual conductance from foliage and bark under rising VPD, are large and critical challenges (McDowell et al., 2022).

A new generation of observations should ideally test the many potential mechanisms underlying hydraulic failure and carbon starvation (Table 2), such as the individual and combined impacts of hypoxia and salinity on root hydraulics, xylem embolism, crown leaf area, and shifts in carbon demand such as for osmoregulation and cellular maintenance (Figure 4). To understand the progression and thresholds of hydraulic failure and carbon starvation, and their underlying mechanisms, will require observations of these parameters in dying woody‐plants in the field; it could also be investigated using ‘point‐of‐no‐return’ studies that measure the key mechanisms during and after relief of hypoxia and salinity, including stress to the point of mortality (e.g. Hammond et al., 2019; Sapes et al., 2021). Partitioning the interdependency of hydraulic failure and carbon starvation will require studies that manipulate the likelihood of one or the other process, for example CO_2_ and light starvation to deplete carbohydrate stores, while simultaneously pushing plants to mortality through anomalous inundation (Hartmann et al., 2013; Quirk et al., 2013; Sevanto et al., 2014). Finally, inundation with and without elevated salinity should be conducted to tease apart the relative roles of hypoxia and salt on mortality mechanisms.

Increasing our understanding of coastal woody‐plant mortality requires not only advanced measurements but also model development and testing against measurements (Collier et al., 2018; Dietze et al., 2018; Medlyn et al., 2015). Models of coastal woody‐plant mortality are in their infancy, but significant potential exists for rapid improvement in their mechanistic realism. Models of sea‐level driven marsh evolution include feedbacks between flooding frequency and duration, plant growth, and sediment accretion (Fagherazzi et al., 2012; Kirwan et al., 2010; Schile et al., 2014). However, attempts to couple marsh models with retreating coastal forests still rely on an assumption of passive retreat, where forest mortality depends on the static inundation of topography, with no tree physiology or groundwater dynamics (Carr et al., 2020; Kirwan et al., 2016). In contrast to the coastal models, models of upland woody plant mortality include both hydraulic failure and carbon starvation, allowing representation of the feedbacks between drivers, mechanisms, and processes underlying mortality (e.g. Christoffersen et al., 2016; Fisher et al., 2010; Koven et al., 2020; McDowell, Fisher, et al., 2013).

Both coastal and upland models lack many of the mechanisms associated with shoreline woody‐plant mortality, such as osmotic constraints on transpiration or salt toxicity to photosynthesis. Therefore, regardless of the modeling approach, new development is required to increase the predictive certainty in our model outcomes. We propose model development for coastal woody‐plant mortality should take advantage of the existing model structures that consider topography while adding the critical drivers and physiological mechanisms of mortality (Figures 3 and 4). The responses to both hypoxia and salinity should be incorporated to allow for simulations of both fresh and saline systems. In addition, such models should include plant responses to climate, such as to rising CO_2_ and VPD, to capture the multiple impacts of both changing water inundation and changing climate (Figure 6).

Given the rapid rate of change not only in water levels but also in atmospheric CO_2_ concentrations, drought, and VPD (IPCC 2021), it is imperative that both measurement and modeling studies of inundation‐induced mortality be conducted within the context of changing climate (Figure 6; Lovelock et al., 2016; Reef et al., 2015). In upland systems, rising CO_2_ can either increase or decrease the likelihood of mortality, rising VPD increases the likelihood of mortality, and their combination may balance the likelihood of mortality (McDowell et al., 2022). We have very limited knowledge of their interactive physiological impacts when combined with increasing hypoxia and salinity, and the impacts of their combinations on mortality *per se* have not been studied. Application of both empirical and numerical experiments can yield significant new insights into the mechanisms of mortality through testing the hypotheses outlined here.

## 9 CONCLUSIONS

Woody plant mortality and ghost forest formation are global phenomena in both fresh and seawater systems (Figure 2). Our understanding and predictive capacity of coastal woody‐plant mortality are both in their infancies; however, the abundance of information from studies of hypoxia and salinity impacts on plants, along with the increasing research focus on ghost forests globally, can enable leaps forward towards better understanding. We hypothesize that both hydraulic failure and carbon starvation emerge from the short and long‐term impacts of hypoxia and salinity on plant water uptake, stomatal closure, photosynthetic biochemistry, phloem transport constraints, and crown loss (Figures 3 and 4). The risk of hydraulic failure precedes that of carbon starvation as determined by the rate of embolism formation relative to the rate of declining carbohydrate content, and their respective thresholds for cytrorrhysis. Feedbacks of reduced growth on mortality appear likely, with decreased growth leading to increased likelihood of future inundation, xylem embolism, and reduced whole‐plant photosynthesis (Figure 4). The mortality processes can be strongly influenced by climatic changes including global rises in CO_2_ and VPD, and such interactions should be considered in future research on ghost forest formation (Figure 6). Modeling mortality processes and their interactions with hypoxia, salinity, and climate change has not yet been attempted, but advances in our knowledge and modeling capabilities of both upland and marsh ecosystems can be leveraged to improve our predictive capacity of ghost forest formation. Ultimately the predictions of well‐tested models can have a substantial influence on society and on development of mitigation strategies to protect coastal woody‐plant ecosystems.

## AUTHOR CONTRIBUTIONS

NGM conceived and led manuscript development and writing. All authors contributed to the literature review and writing of the manuscript.

## Data Availability

Data sharing is not applicable to this article as no new data were created or analyzed in this study.
